# Dairy farmers’ knowledge, attitudes, and practices regarding the brucellosis surveillance and control program in Bogor, Indonesia

**DOI:** 10.14202/vetworld.2023.126-133

**Published:** 2023-01-19

**Authors:** Heris Kustiningsih, Etih Sudarnika, Chaerul Basri, Mirnawati Sudarwanto

**Affiliations:** 1 Animal Health Training Center, Ministry of Agriculture Republic Indonesia, Cinagara, Bogor 16740, Indonesia; 2 Animal Biomedical Sciences Study Program, Postgraduate School, IPB University, Bogor 16680, Indonesia; 3 Department of Animal Disease and Veterinary Public Health, School of Veterinary Medicine and Biomedical Science, IPB University, Bogor 16680, Indonesia

**Keywords:** brucellosis control, dairy cattle, knowledge, surveillance

## Abstract

**Background and Aim:**

Brucellosis is an infectious and zoonotic disease that affects people’s health and the economy in most countries. Brucellosis is still prevalent in several Indonesian regions. This study aimed to analyze the correlation between the characteristics, knowledge, attitudes, and practices (KAP) of dairy farmers in Bogor District in supporting brucellosis control and surveillance programs.

**Materials and Methods:**

The study was cross-sectional. Data were collected through interviews with 151 dairy farmers in Bogor Regency, West Java, Indonesia. The outcome is brucellosis surveillance and control practice among dairy farmers, and the variables include individual characteristics, knowledge, and attitudes toward brucellosis surveillance and control. Descriptive analysis and path analysis were used in statistical analysis.

**Results:**

The majority of farmers’ knowledge, attitudes and practices were moderate, with the percentages 67.55%, 60.92%, and 41.72% respectively. Formal education, training, and dairy rising length are variables that have a direct and significant impact on knowledge level. Knowledge is the variable that influences the overall level of attitude. Age, knowledge, and attitude are factors that influence the practice of brucellosis surveillance and control.

**Conclusion:**

Although the practice level of brucellosis surveillance and control for dairy farmers in Bogor Regency is moderate, efforts to improve it are still required. The basic effort is critical for increasing farmers’ knowledge.

## Introduction

Brucellosis is an infectious and zoonotic disease caused by *Brucella* spp. Four species of *Brucella* live in animals that can infect humans, namely *Brucella abortus* in cattle, *Brucella melitensis* in goats and sheep, *Brucella suis* in pigs, and *Brucella canis* in dogs [1]. Brucellosis in cattle during animal production causes huge economic losses due to clinical disease, abortion, neonatal loss, increased calving interval, decreased fertility, decreased milk production, increased culling rate due to metritis, and emergency slaughter of infected animals [2, 3]. The eradication of brucellosis in animals is an important step in controlling human disease [4]. Brucellosis in humans causes periodic fever, muscle pain, and reproductive disorders, namely, epididymitis, impaired spermatogenesis in males, and early-trimester abortion in females [5, 6]. Brucellosis harms the health and economy of people in most countries. Brucellosis in Indonesia harms the livestock economy. Basri and Sumitro [7] state that large ruminant livestock in Indonesia is estimated to suffer losses of up to 3.6 trillion Indonesian rupiah per year or 1.8% of the total value of livestock assets. The control program for eradicating brucellosis in Indonesia started in 1996/1997 through a vaccination program and conditional slaughter (test and slaughter) [8]. The Indonesian Directorate General of Livestock and Animal Health 2013 issued a National Brucellosis Eradication Roadmap in Indonesia. However, the roadmap has not been implemented optimally, so some areas still report the prevalence of brucellosis in the high category (>2%). According to Balai Veteriner Subang [9], the prevalence of brucellosis was 3.6% in West Java, 5.10% in West Bandung District [10], and 15.77% in Bandung District [11]. As a result, efforts must be made to optimize the implementation of the brucellosis eradication roadmap.

Involving the community is one way to improve the implementation of the brucellosis eradication roadmap (farmers). Cooperation between community and animal health authorities is one method for controlling and eradicating brucellosis [12, 13]. Community participation in the brucellosis control program can foster a sense of program ownership and position the community as the program’s subject or actor. The community has disease related experience, knowledge, and skills. This potential can be used to help control the disease [14].

The community’s (farmers’) potential must be maximized so that they can play a role in brucellosis control. Efforts to increase farmers’ potential can be made through education and training. However, before determining the appropriate training and education, farmers’ knowledge, attitudes, and practices (KAP) regarding brucellosis control and surveillance in the field must be assessed.

This study aimed to analyze the correlation between the characteristics and KAP of dairy farmers in Bogor District in supporting brucellosis control and surveillance programs.

## Materials and Methods

### Ethical approval

The Human Research Ethics Committee of Bogor Agricultural University approved this research proposal under the number: 623/IT3.KEPMSM-IPB/SK/2022.

### Study period and location

The study was conducted from May to July 2022 in the dairy cattle pouch areas of Bogor Regency, namely Cisarua, Ciawi, Megamendung, Pamijahan, Cilebut, and Tajurhalang sub-districts. The area is in the western, northern, and southern parts of Bogor Regency, West Java, Indonesia. Bogor Regency covers an area of 2,663.81 km^2^. Bogor Regency has the fourth-largest dairy cattle population in West Java and the second-largest dairy cattle population in Indonesia, with 8739 heads. Bogor Regency’s geographical location is quite strategic in supporting the need for animal-derived food because it is close to Indonesia’s capital city.

### Study design

**Figure-1:**
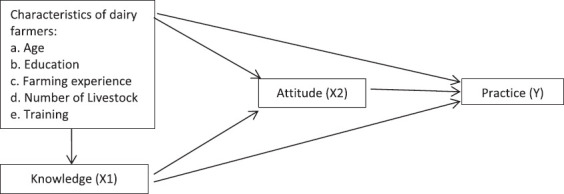
Research framework on knowledge, attitudes, and practices of dairy farmers on brucellosis surveillance and control in Bogor, Indonesia.

This was a cross-sectional study with a KAP survey, as well as interviews and observations of dairy farmers. The variables in this study were the characteristics, KAP of brucellosis surveillance and control in Bogor Regency dairy farmers. Sex, age, last education, dairy rising length, breeding purpose, number of dairy cattle owned, and number of training completed are all characteristics. Farmers’ KAP regarding brucellosis control includes causative agent, mode of transmission, clinical symptoms, prevention, control, and surveillance. Figure-1 depicts the research framework in summary.

### Sample size and population

Bogor Regency’s population was dairy farmers, with a farmer household research unit. Farmers’ respondents came from Pamijahan, Cisarua, Megamendung, Ciawi, Cilebut, and Tajurhalang sub-districts representing the western, southern, and northern regions of Bogor Regency. Farmers were interviewed about KAP related to brucellosis surveillance and control programs. Sampling used simple random sampling with an assumption of 26% prevalence, 7% error, and 95% confidence level, using WinEpi software (Ignacio de Blas. Facultad de Veterinaria, Universidad de Zaragoza ©2006; http://www.winepi.net), the number of samples was 151 farmers [15].

### Data collection

Data were gathered through direct interviews using a structured questionnaire. The questionnaire, which included up to 80 questions, was designed to investigate farmers’ KAP in the brucellosis surveillance and control program. The questionnaire was tested for validity and reliability to ensure its feasibility before being used. The farmer’s knowledge level questionnaire was designed with 20 questions regarding brucellosis surveillance and control. Respondents were given three answers: True, false, and do not know [16]. For knowledge, the category of “good” level if the respondent’s answer score reaches >14, the level of “sufficient” if the respondent’s answer score was 7–14, and the level of “less” if the respondent’s answer score was <7. The farmer’s attitude level assessment was designed using a Likert Scale of 20 questions, with answers strongly agree, agree, disagree, and strongly disagree. The attitude category is the “good” level if the respondent’s answer score reaches >40, the “adequate” level if the respondent’s answer score was 20–40, and the “less” level if the respondent’s answer score was <20. The farmer practice assessment includes 40 questions divided into prevention practices (20 questions), control practices (14), and reporting practices six questions. The total score was 60, with a good level category for scores was greater than 40, a moderate level category for scores 20–40, and a low level category for scores <20.

### Statistical analysis

Based on the standardized Pearson correlation coefficient, the data were analyzed using pathway analysis to estimate the magnitude of each variable’s direct and indirect effect.

## Results

### Dairy farmers’ characteristics

Age, education, length of breeding, number of dairy cattle, and the amount of training received were among the characteristics of dairy cattle farmers observed as research variables (Table-1).

**Table-1:** Bogor Regency dairy farmers’ characteristics.

Characteristic	Frequency	Percentage
Age		
<25 years old	8	5.3
25–50 years old	104	68.9
>50 years old	39	25.8
Education		
Non-educated	14	9.3
Elementary school	32	21.2
Middle school	60	39.7
High school	26	17.2
D1/D2/D3	4	2.0
S1/S2	16	10.6
Dairy rising length		
<5 years	63	41.7
5–10 years	43	28.5
>10 years	45	29.8
Dairy cattle owned		
<5 cattle	34	22.5
5–10 cattle	66	43.7
>10 cattle	51	33.8
Training		
Never	60	39.7
Once	64	42.4
Twice	22	14.6
Three times	5	3.3

Most of the respondents in this study were 25–50 years old. The age in this range is the productive age. The level of formal education according to the farmer’s last diploma, and the largest is with junior high school education (60%), then elementary school level (21.2%); there are even non-schooled farmers (9.35%). The level of experience or length of farming is mostly more than 5 years, reaching 58.3%. The number of dairy cattle owned by farmers is mostly between 5 and 10. Some farmers have attended training once (42.15%), but 39.7% have never attended any training.

### Dairy cattle farmers’ KAP

The KAP of respondents regarding brucellosis surveillance and control was assessed by assigning a value/score to each variable. The majority of participants fell into the moderate category in terms of KAP. The percentages were (67.55%), (60.92%), and (41.72%), respectively. Table-2 contains the specifics.

**Table-2:** Knowledge attitude and practice assessment of dairy farmers in Bogor Regency.

Characteristics	Frequency	Percentage
Knowledge		
Good	25	16.56
Moderate	102	67.55
Poor	24	15.89
Attitude		
Good	54	35.77
Moderate	92	60.92
Poor	5	3.31
Practice		
Good	54	35.76
Moderate	63	41.72
Poor	34	22.52

### The relationship between personality and KAP

Path analysis calculates the correlation and magnitude of each variable’s direct and indirect effects. Path analysis demonstrates the relationship between independent and dependent variables influencing KAP. The research conceptual framework serves as the foundation for the structural equation model. Table-3 shows three different models.

**Table-3:** Regression equations were used in the research path analysis of dairy farmer’s knowledge, attitudes, and practices regarding brucellosis surveillance and control in Bogor Regency.

Model	Non-independent variable	Independent variable	Structural equation
Model 1	X1	a, b, c, d, e	X1 = r_X1_a + r_X1b_b + r_X1c_c + r_X1d_d + r_X1e_e + r_X1_*ɛ X1
Model 2	X2	a, b, c, d, e, X1	X2 = r_X2_a + r_X2b_b + r_X2c_c + r_X2d_d + r_X2e_e + r_X2X1_X1 + r_X2_*ɛ X2
Model 3	Y	a, b, c, d, e, X1, X2	Y = r_Ya_a + r_Yb_b + r_Yc_c + r_Yd_d + r_Ye_e + r_YX1_X1 + r_YX2_X2 + ry*ɛ Y

a=Age, b=Education, c=Dairy rising length, d=Dairy cattle total, e=Training, X1=Knowledge, X2=Attitude, Y=Practice, ρij=Path coefficient, ɛi.=Residual error

**Figure-2:**
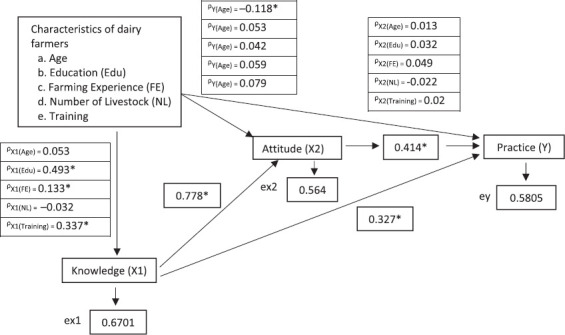
Value of path coefficients for research variables of knowledge, attitudes, and practices of dairy farmers on brucellosis surveillance and control in Bogor Regency. (ex1: residual; ex2: residual x2 dan ey: residual y; *significant effect).

Based on the research concept framework, the path coefficient value indicates the correlation between variables. Figure-2 shows the path coefficient values for each independent and dependent variable based on the structural equations in Table-2.

The farmers’ characteristics that directly influence the level of knowledge are the variables of age, length of raising, and training, with path coefficients (r) of 0.493, 0.133, and 0.337, respectively. There are no farmers’ characteristics that have a direct influence on attitudes. However, the level of knowledge has a direct and significant effect on the level of attitudes, with a path coefficient (r) of 0.778. Age, knowledge level, and attitudes directly influence the practice level, with path coefficients (r) of −0.118, 0.327, and 0.414, respectively. The path coefficient values of each independent and dependent variable are fully presented in Figure-2.

### Individual characteristics and knowledge correlation (Model 1)

Table-4 shows the path coefficient values of the direct influence of individual characteristics on dairy farmers’ knowledge of brucellosis surveillance and control in Bogor Regency.

**Table-4:** Direct and indirect effects, as well as the significance of variables influencing farmer knowledge of brucellosis surveillance and control in dairy cattle in Bogor Regency.

Variable effect	Direct effect	Total effect	%	Sig
a toward X1	0.053	0.053	2.787	0.412
b toward X1	0.493	0.493	25.92	0.000*
c toward X1	0.133	0.133	6.99	0.041*
d toward X1	0.032	0.032	1.68	0.587
e toward X1	0.337	0.337	17.71	0.000*
Total	1.048			
	(55.10%)		55.10	

a=Age, b=Education, c=Dairy raising length, d=Dairy cattle owned, e=Training, X1=Knowledge, X2=Attitude, Y=Practice,

* Indicates a significant correlation at=0.05, confidence interval 95%

The effect of formal education on farmers’ knowledge related to surveillance and control of brucellosis has the highest percentage compared to other characteristics. The real influence of formal education described by model 1, which is 25.92% from 55.10% or approximately 47.04%, is influenced by formal education. Farmers’ formal education also influences their knowledge of brucellosis surveillance and control based on the results of the partial test of characteristics. Training and length of dairy raising are two other factors that influence brucellosis surveillance and control knowledge.

### Individual characteristics, attitude, and knowledge correlation (Model 2)

Individual characteristics and knowledge, according to the research concept framework, influence attitude both directly and indirectly. Table-5 shows the path coefficient values for the direct and indirect influence of individual characteristics and knowledge variables to the attitude of dairy farmers in Bogor Regency toward brucellosis surveillance and control.

**Table-5:** The direct and indirect effects, as well as the significance of variables influencing farmer’s attitudes toward brucellosis control and surveillance in dairy cattle in Bogor Regency.

Variable effect	Direct effect	Indirect effect through X1	Total effect	%	Sig
a toward X2	0.013	0.041	0.054	2.62	0.807
b toward X2	0.032	0.384	0.416	20.05	0.622
c toward X2	0.049	0.103	0.152	7.36	0.382
d toward X2	−0.022	−0.249	−0.271	−13.07	0.652
e toward X2	0.020	0.262	0.282	13.62	0.729
X1 toward X2	0.778		0.778	37.54	0.000*
Total	0.87		1.411		
	(68.10%)			68.1	

a=Age; b=Education; c=Dairy raising length; d=Dairy cattle owned; e=Training; X1=Knowledge; X2=Attitude; Y=Practice;

* Indicates a significant correlation at=0.05; confidence interval 95%.

Knowledge was a significant variable and had the greatest influence on the attitude of dairy farmers. Table-5 show that knowledge had a direct effect of 37.54% of the total 68.1% of all variables, or in other words the contribution of the influence of the knowledge was 55% of the contribution of all variables.

### Individual characteristics, knowledge, attitude, and practice correlation (Model 3)

Individual characteristics, knowledge, and attitudes influence practices directly and indirectly. The path coefficient values of direct and indirect influence of individual characteristics, knowledge, and attitudes toward the practice of dairy farmers related to brucellosis surveillance and control in Bogor Regency are presented in Table-6.

**Table-6:** The direct and indirect effects, as well as the importance of variables influencing farmers’ practices in the control and surveillance of brucellosis in dairy cattle in Bogor Regency.

Variable effect	Effect				Indirect total	Total effect	%	p-value

	Direct	Indirect						

		X1	X2	X1*X2				
a toward Y	−0.118	0.053	0.013	0.017	0.083	−0.035	−0.87	0.033*
b toward Y	0.053	0.493	0.032	0.159	0.684	0.737	18.40	0.442
c toward Y	0.042	0.133	0.049	0.043	0.225	0.267	6.66	0.455
d toward Y	0.059	0.032	−0.022	0.010	0.020	0.079	1.98	0.231
e toward Y	0.079	0.337	0.020	0.109	0.466	0.545	13.60	0.183
X1 toward Y	0.327	-	0.322	-	0.322	0.649	16.21	0.001*
X2 toward Y	0.414	-	-	-	-	0.414	10.34	0.000*
Jumlah	0.856	1.048	0.414	0.338	1.800	2.66		
	(21.37%)	(26.16%)	(10.34%)	(8.43%)	(44.93%)		66.3	

a=Age, b=Education, c=Dairy raising length, d=Dairy cattle owned, e=Training, X1=Knowledge, X2=Attitude, Y=Practice,

* Indicates a significant correlation at=0.05, confidence interval 95%

Variables (age, knowledge, and attitudes) significantly affect farmers’ practice of surveillance and control of brucellosis compared to other characteristics. The influence of formal education is 18.40% from 66.30% or approximately 27.74%. The farmers’ formal education has the largest percentage of total influence. Although it does not show a significant correlation to practice, the magnitude of the total influence is due to the large indirect influence obtained from attitudes, knowledge, and age.

## Discussion

The majority of Bogor Regency’s dairy farmers are between the ages of 25 and 50. This age range is the productive age, which can help with the implementation of dairy cattle in terms of energy, innovation, and work spirit. However, when it comes to formal education, most farmers have a medium to low level of education, with the majority being unschooled (70.20%). About 91.14% of respondents have been raising dairy cattle for more than 3 years. This information indicates that the farmer has a long history of raising dairy cattle.

The level of education of a farmer influences their perception. Education influences farmers’ ability to act correctly [17]. Formal education is important because it influences farmers’ ability and proclivity to learn more [18]. Other research has found that farmers with lower levels of education are less knowledgeable about brucellosis [19].

The knowledge of farmers related to surveillance and control of brucellosis is sufficient and low at 83.44%. Efforts are needed to increase farmers’ knowledge, for example, through education and training [20]. The level of attitude of farmers regarding surveillance and control of brucellosis in the sufficient and low categories is 64.23%. The level of practice is also in the sufficient and less category of 64.24%. Farmers’ KAP regarding brucellosis surveillance and control must be improved until they reach a good category.

Knowledge of farmers in this study is directly influenced by formal education, dairy raising length, and training. The farmers’ knowledge could change and develop according to their abilities, needs, experience, and level of acceptance of information in the surrounding environment. In the opinion of Kheiri *et al*. [21], education increases knowledge; this is in line with the results of this study that there is a significant correlation between education and knowledge [22]. Education is the formation of healthy behavior for individuals and others in the future. Behavior that is regulated through education aims at conditioning, such as deepening, training, and practice [23]. The training that farmers attend has a direct influence on their knowledge. The more often farmers attend training, their knowledge will increase. The correlation between knowledge and training in this study is in line with Niati *et al*. [24] argument that training is a process of teaching certain knowledge, skills, and attitudes so that individuals are more skilled and able to carry out their responsibilities better according to standards. Farmers have attended more and more training related to brucellosis. A persons’ experience is extremely valuable in preparing for future challenges. People who are experienced in specific jobs will have more knowledge than those who are inexperienced. This study found that farmers’ knowledge is directly and significantly influenced by their experience raising dairy cattle.

Farmers’ attitude in this study was directly influenced by knowledge of 37.54% from 68.10% or approximately 55.12%, in line with the results of research by Luttrell and Sawicki [25] that knowledge is the basis for the formation of one’s attitude. Liobikienė and Poškus [26] also revealed that knowledge could change a person’s beliefs and values. A person’s knowledge will encourage them to form a belief that will affect attitudes. The existence of a real correlation between knowledge and attitudes in this study means that the higher the knowledge, the better farmers’ attitudes regarding brucellosis surveillance and control. In addition to knowledge, formal education and training variables indirectly affect farmer attitudes. However, the relationship is not significant (significant). Skills and expertise can be obtained directly through education/knowledge or indirectly through experience. This knowledge and experience ultimately shape a person’s perception of something. Hence, it has a natural effect on attitudes in line with the opinion of Hesaraki *et al*. [27], which explains that attitudes can be formed from knowledge through learning or experience.

The total effect of various variables that affect the practice described by model 1 is 66.3%. Education, knowledge, and training are the three variables that influence the practice of surveillance and control of brucellosis. Education and training have the largest total influence on practice. However, they do not show a significant correlation to practice; the magnitude of the total influence is due to the large indirect effect of attitudes and knowledge. Education and training have a direct and significant impact on farmers’ knowledge. Age and attitude are the variables that significantly influence knowledge, even though their total value of the effect is lower than education and training in practice.

Based on the research path analysis results, three paths significantly influence practice: First through attitude. Attitudes have a direct and significant effect on practice, indicating that the better the attitude of the farmers, the better the level of practice in brucellosis surveillance and control. This study’s results align with research by Noviana *et al*. [28], that the better and more positive the attitude of the breeding kennel owner is, the better the level of practice in controlling this disease will be. Likewise, Wicaksono’s statement [29] that the more positive the attitude of the traders, the better the biosecurity practices they apply. According to Sutanto [30] and O’Kane *et al*. [31], attitudes that arise can be formed from beliefs, feelings, or judgments on prevention and control practices followed by behavioral tendencies. A person’s attitude can change due to the influence of social interaction. In social interaction, there is a mutually influencing correlation between individuals with one. Individuals react to form certain patterns of attitudes toward various objects encountered, and these attitudes can trigger easier repairs.

The second path of variables that influence practice is knowledge. Knowledge influences practice indirectly through attitudes. In this case, attitude affects the practice of what is learned and known. Practice is formed through a path that starts from an attitude influenced by the level of knowledge, while knowledge is influenced by education, dairy raising length, and training. This second path describes practices shaped by outside influences due to habit or imitating the practices of someone they thought of as a role model. The results of path analysis are supported by the opinion of Walgito [32], which states that human behavior is formed and learned. This study’s significant correlation between attitudes toward practice aligns with Kruglanski *et al*. [33], which states a strong correlation between attitudes and actions.

The age variable affects practice directly and significantly with a path coefficient (r) of −0.118. Age affects attitudes directly with a negative path coefficient value, indicating that the older the respondent, the more negative the attitude. The old farmers’ respondents in this study have a low educational background, so it is difficult to change their insights. Older farmers still practice the old ways that do not attach importance to the practice of surveillance and control of brucellosis. Farmers think that the practices they have done are correct and easy. Meanwhile, young farmers are more interested in following the latest information developments so that their knowledge of young farmers increases and is encouraged to practice based on the knowledge gained in this case related to surveillance and control of brucellosis. The result is in line with the opinion of Petruzzelli *et al*. [34] that younger people tend to be more open to new information, ideas, and broader knowledge.

Formal education and training of farmers on the practice of surveillance and control of brucellosis based on pathway analysis in this study had the second and third-largest total effects. However, it has an insignificant (significant) effect. The total effect is largely due to the indirect effect of knowledge and attitudes. This result exhibits the important contribution of formal education and training to practice brucellosis surveillance and control by farmers. This significant contribution can be used as an evaluation tool to improve the quality of human resources available to farmers in the field. According to Awwad *et al*. [35], education level was an important and positive supporter of the implementation of brucellosis prevention practices. Khan *et al*. [36] also stated that the level of education and knowledge of dairy farmers positively correlated with disease prevention practices in the field.

There is a positive relationship between education and knowledge, knowledge and attitudes, and attitudes toward practice. A positive correlation indicates that the more valuable knowledge and attitudes are, the better the practice. The knowledge variable had the greatest impact on the level of practice obtained through direct and indirect influences. According to the path analysis results, the total effect of all research variables in this study, including characteristics, knowledge, and attitudes of farmers toward brucellosis surveillance and control practices, was 66.30%. Other factors not included in the study influenced 33.70% of the participants.

Improving dairy farmers’ practice of surveillance and control of brucellosis can be done through training. Training is an important factor in increasing the knowledge and skills of farmers in brucellosis surveillance and control so that farmers who have attended training can apply the theory and practice they have acquired [37]. Training is a factor that can influence practice. In addition, the training also aims to improve workers’ knowledge, skills, performance, and attitudes. According to Sarı [38], training should be provided before someone begins working on obtaining the information they require in order to do their job effectively and safely.

## Conclusion

The KAP of dairy farmers in Bogor Regency regarding brucellosis supervision and control are adequate. According to the research’s correlation pattern between variables, farmer practice is directly influenced by age, knowledge, and attitudes. Knowledge has a direct and significant influence on attitudes, and knowledge has a direct and significant influence on formal education, training, and experience in dairy cattle raising. Efforts must be made to improve farmers’ practices so that they can be classified as good. The fundamental effort to increase farmer knowledge and the appropriate intervention is to conduct training.

## Authors’ Contributions

HK, ES, CB, and MS: Conceived the idea. HK, ES, and CB: Designed the study, developed the theory and prepared the tools and materials. HK: Collected the data. HK and ES: Analyzed the data. HK and ES: Wrote the manuscript with input from all authors. All authors discussed the results and contributed to the final manuscript, provided critical feedback, and helped shape the research, analysis, and manuscript. All authors have read and approved the final manuscript.
